# Supplementary material for: Effect of Gravity on Bacterial Adhesion to Heterogeneous Surfaces
Source: Pathogens. 2023 Jul 15;12(7):941. doi: 10.3390/pathogens12070941 (PMC10383686; doi:10.3390/pathogens12070941)
Supplement: Supplementary file 1 [file pathogens-12-00941-s001.zip › Hogan Supplementary Data v2.pdf]

## Supplementary Data

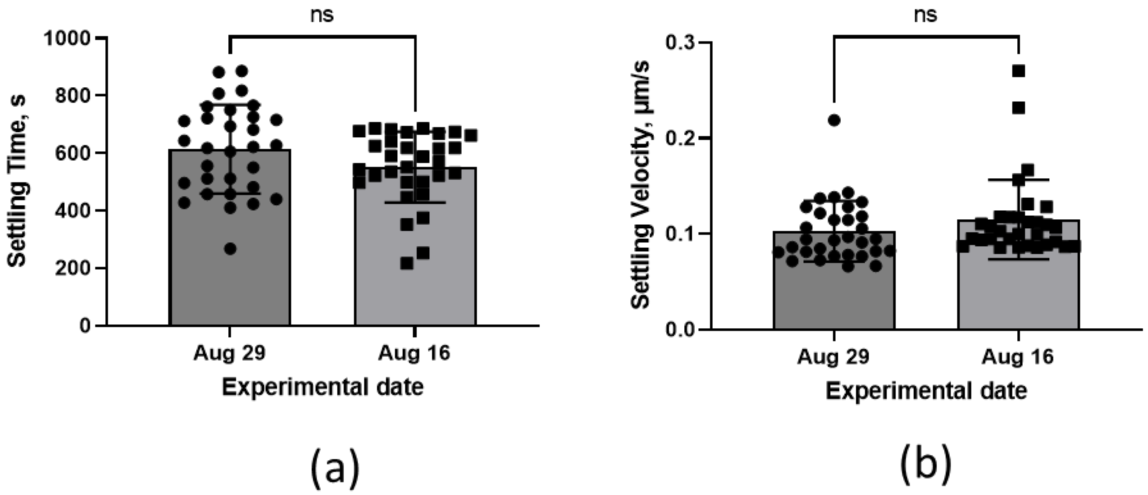


**Supplementary Figure S1. Bacterial gravitational setting velocity.** Settling velocity was estimated by measuring the length of time for 31 bacteria to drop from the top of a 59 µm high chamber (after flipping a chamber on which bacteria were settled on the bottom) to the bottom, as determined by waiting for each bacterium to come into focus using phase contrast video microscopy focused on the lower surface. **(a)** Likely due to variations in size, bacteria displayed a distribution of settling times. The mean time was 614 s on the first day the experiment was performed, and 551 s on the second day. **(b)** The velocity of each bacterium was determined by dividing 59 µm by the settling time and determined to be 0.103 on the first day and 0.115 on the second day. The symbol "ns" indicates no significant difference between the measured value for the two days, as determined by an unpaired t-test assuming the same standard deviations. All 62 bacteria over the two days were therefore used to estimate the settling velocity given in the methods.

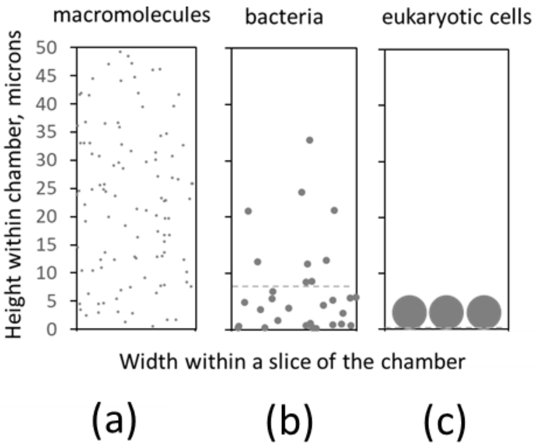


**Supplementary Figure S2. Bacterial transport is at the interface of two regimes with respect to gravitational settling**. In the absence of flow, particles form an 'atmosphere' in which the concentration decays exponentially with a characteristic height $h_c = D/V_g$ from bottom, where $D$ is the diffusion coefficient and $V_g$ is the sedimentation velocity of the particles. Both $D$ and $V_g$ can be estimated from the size and density of the particles in order to predict the characteristic height and thus the distribution of particles at equilibrium[1]. To illustrate this phenomenon in this figure, the position of particles is determined from a random distribution of x-positions, and an exponential distribution of y-positions so that $p(y) \propto \exp\left(-\frac{y}{h_c}\right)$. **(a)** A large macromolecule of radius 15 nm and density 1.3 g/ml would sediment to a characteristic height $h_c = 2\ cm$. This is much larger than the height of a microfluidic chamber, so diffusion dominates over sedimentation. **(b)** In contrast, a bacterium of radius 0.5 μm and density 1.1 g/ml, would sediment to a characteristic height $h_c = 7\ \mu m$ from the surface, which is comparable to the size of the bacteria, so sedimentation and diffusion are both significant. **(c)** A eukaryotic cell of radius 3 μm and density 1.05 g/ml would settle to a characteristic height $h_c = 75\ nm$ from the surface, which is much smaller than the size of the cells, so sedimentation dominates over diffusion. Thus, diffusion dominates for macromolecules and sedimentation dominates for typical eukaryotic cells, while both processes impact bacterial transport. This analysis suggests while the ratio of characteristic height to chamber height determines whether sedimentation is important, a more complex nondimensional constant is needed to describe when diffusion is important. We define the "sedimentation constant" as the ratio of the particle diameter to the characteristic height, which we will call:

$$Sc = \frac{2r}{h_c} = \frac{2rV_g}{D} \tag{S1}$$

When $Sc \gg 1$, diffusion can be neglected, because the particles remain in contact with the surface and sedimentation is likely to dominate over diffusion for bringing new particles in contact with the surface. For example, for the model eukaryotic cells in panel c, $Sc = 6/0.075 = 80$. However, when $Sc \sim 1$, the particles remain in an "atmosphere" above the surface that is determined by the balance of sedimentation and diffusion. For panel b, $Sc = 0.5/8 = 0.12$, and for bacteria between 0.25 and 1.5 um in radius, $Sc$ ranges from 0.01 to 10.

[1] The diffusion coefficient may be estimated from the Boltzman factor $k_B$, temperature $T$, viscosity $\mu$ and particle radius $r$, using the Stokes-Einstein-equation, $D = \frac{k_B T}{6\pi\mu r}$. The sedimentation velocity $V_g$ may be estimated from the gravitational force on a particle $F_g$ using Stoke's law for frictional drag, $V_g = \frac{F_g}{6\pi\mu r}$, while gravitational force can be estimated from Newton's law: $F_g = mg$, where $g$ is gravitational acceleration and $m$ is the mass of the particle less the mass of the fluid it displaces, which for a particle with density relative to fluid $\Delta\rho$, is $m = \frac{4}{3}\pi r^3 \Delta\rho$. Thus, $F_g = \frac{4}{3}\pi r^3 \Delta\rho g$ and the rate of gravitational sedimentation is $V_g = \frac{2r^2\Delta\rho g}{9\mu}$. The characteristic height is then $h_c = \frac{D}{V_g} = \frac{k_B T}{\frac{4}{3}\pi r^3 \Delta\rho g}$.

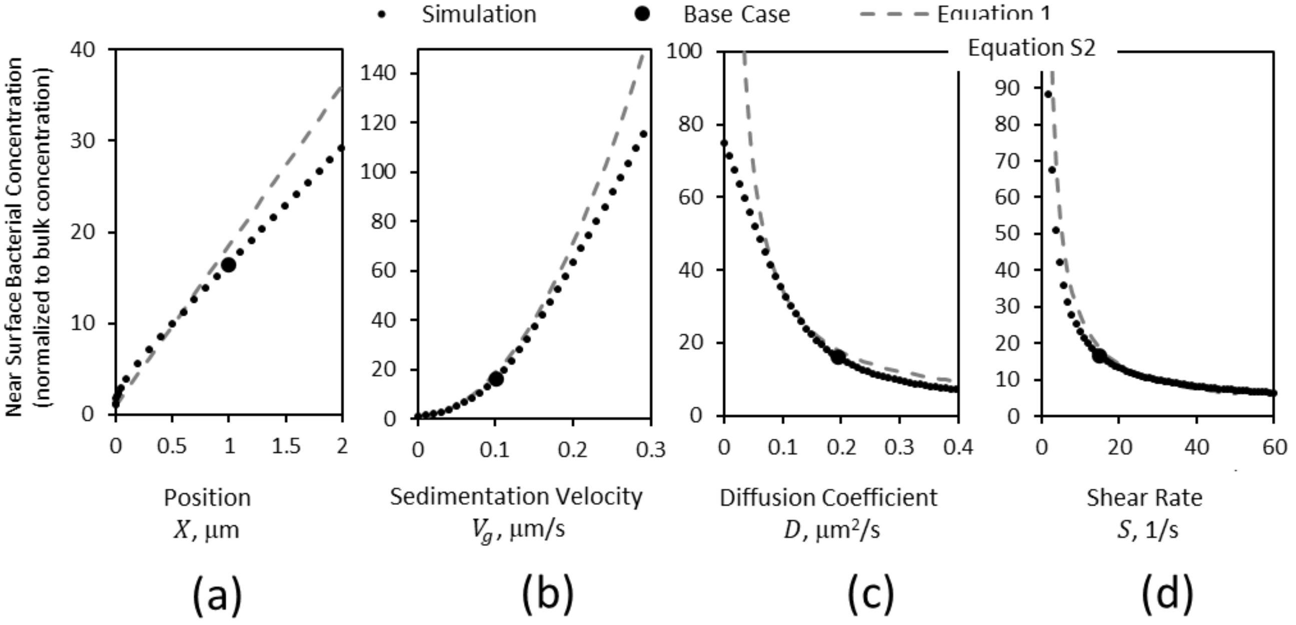


**Supplementary Figure S3. Modeling the effect of experimental conditions on the near-surface concentration of bacteria**. To understand how the concentration depends on distance into chamber, sedimentation velocity, diffusion coefficient and shear rate, we sought to fit the simulation data shown in Figure 3 with a simple algebraic equation for the concentration of bacteria near the surface versus in bulk solution $\frac{C_{surf}}{C_{bulk}}$. The grey dashed lines in the figure show the prediction from the Equation S2 below, where C is a constant that is 0.5 µm$^{-1}$ in these conditions[2].

$$\frac{C_{surf}}{C_{bulk}} = 1 + C\frac{V_g^2 X}{DS} \qquad \text{(S2)}$$

Note that within a range, this simplified model fits the data, showing that the concentration of bacteria near the surface increased linearly with position, increased with the square of the sedimentation velocity, and decreased with the inverse of both the diffusion coefficient and shear rate. However, when the sedimentation velocity is large, or the diffusion coefficient is small, Equation S2 overestimates the degree of sedimentation, because the simulations predict a linear relationship in those conditions. It should be noted that this occurs when $V_g > 0.2$ (**Figure S2b**) or $D < 0.1$ (**Figure S2c**). In both cases, the Sedimentation constant defined in Equation 1 ($S_c = \frac{2rV_g}{D}$) is greater than 1, so gravity brings cells to the bottom without significant mitigation by diffusion. Nevertheless, Equation 2 is fairly accurate when diffusion and gravity are both significant contributors to mass transport, which is the focus of this study. Note that because larger bacteria sediment more rapidly and diffuse more slowly, the effects described here predict that the normalized increase in near-surface concentration ($\frac{C_{surf}}{C_{bulk}} - 1$) will increase very dramatically with bacterial size. For a round bacteria, Equation S2 can be used to show that the normalized increase in concentration is proportional to $r^5$:

$$\frac{C_{surf}}{C_{bulk}} - 1 = \frac{CX}{S}\frac{\left(\frac{2r^2\Delta\rho g}{9\mu}\right)^2}{\frac{k_B T}{6\pi\mu r}} = \frac{\frac{CX}{S}4r^4(\Delta\rho^2 g^2)2\pi r}{3 * 9\mu \quad k_B T} = \frac{8\pi CX\Delta\rho^2 g^2}{27S\mu \quad k_B T}r^5 \qquad \text{(S3)}$$

---

[2] Without more simulations, it is unclear if the value of C as 0.5 µm$^{-1}$ value indicates the importance of the 2.5 um thick layer we averaged to calculate the near-surface concentration to be consistent with the experimental measurements, or the diameter of the bacteria, or something else. However, this is not critical to our conclusions.

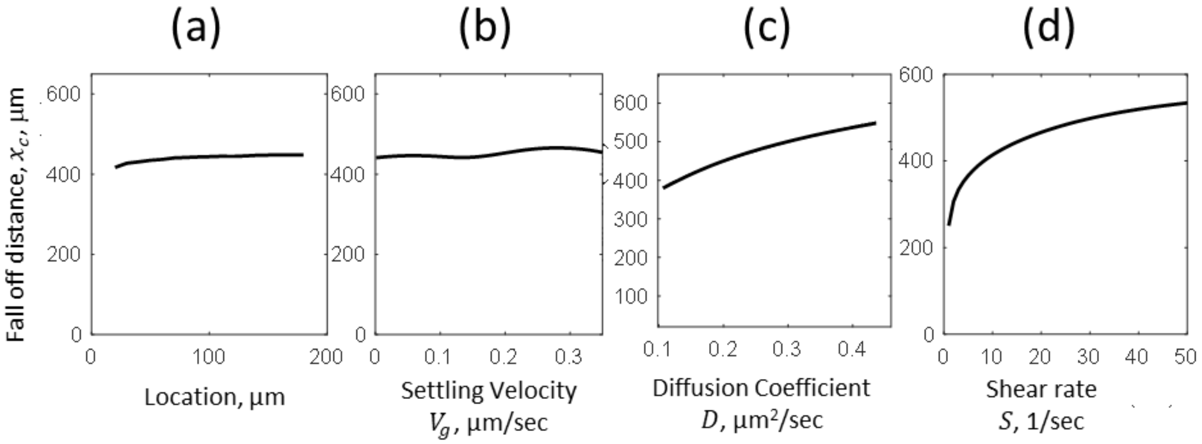


**Supplementary Figure S4. Effect of experimental conditions and bacterial properties on the fall-off distance.** The fall-off distance xc is the characteristic distance at which the density of bound bacteria exponentially decays from the edge density $B_{edge}$ to the interior density $B_{int}$. Simulations were performed with the parameters given in Table 1 except for the parameter indicated in each panel.
