# Supplementary figures and images for: Effect of Gravity on Bacterial Adhesion to Heterogeneous Surfaces
Source: Pathogens. 2023 Jul 15;12(7):941. doi: 10.3390/pathogens12070941 (PMC10383686; doi:10.3390/pathogens12070941)

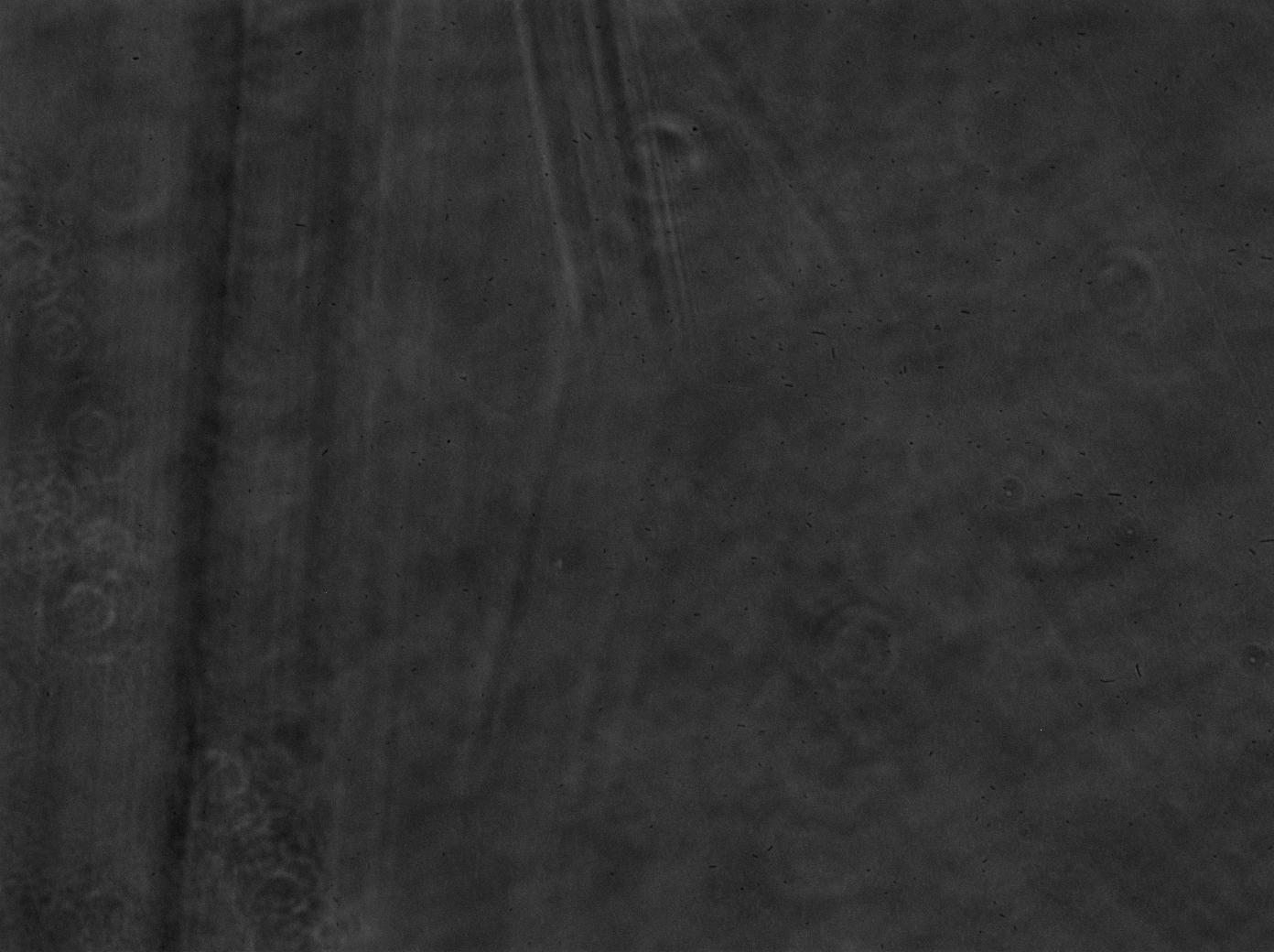

Supplement: Supplementary file 1 [file pathogens-12-00941-s001.zip › S6.Figure6b100ugRb_r2..gif]

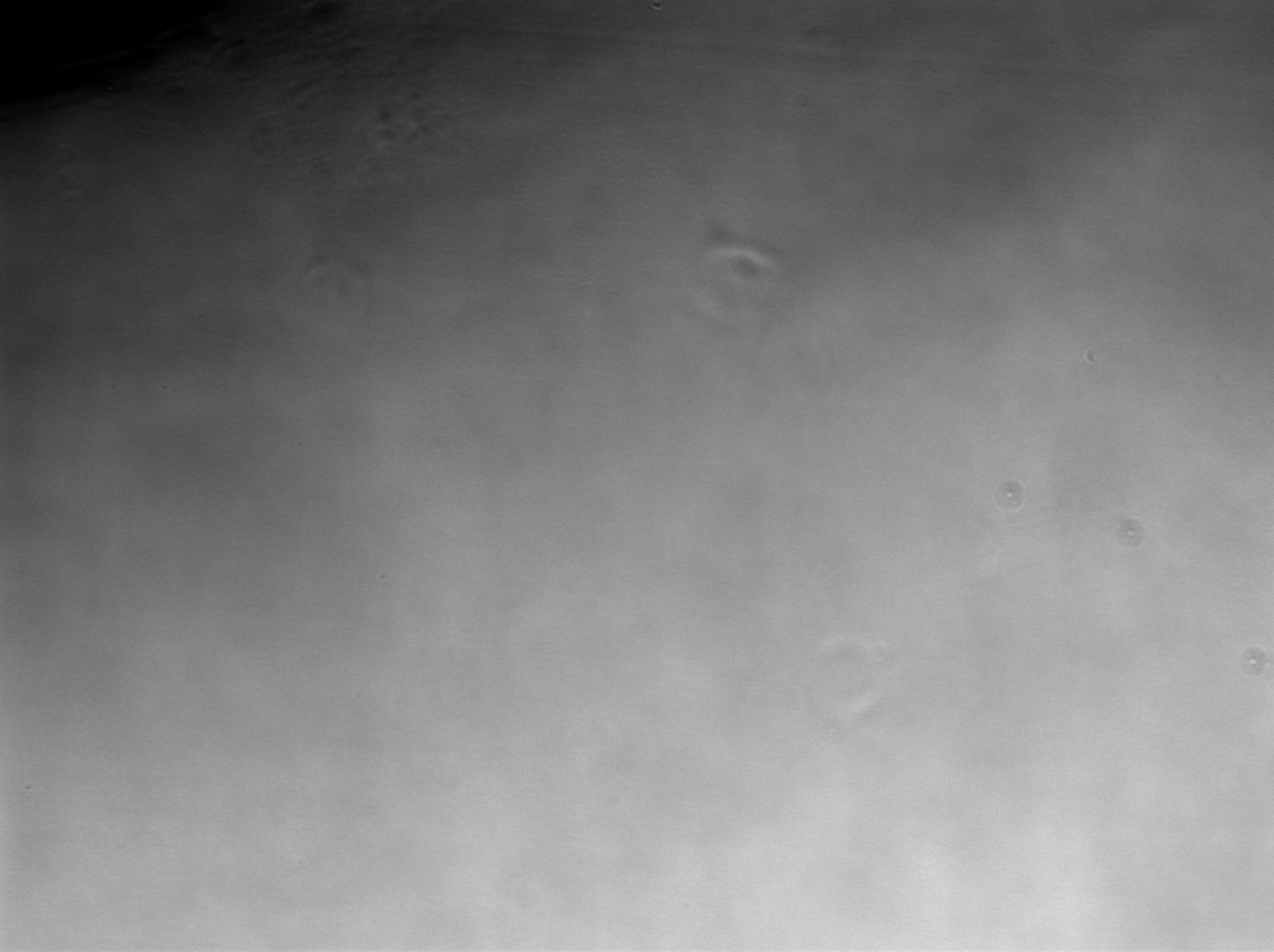

Supplement: Supplementary file 1 [file pathogens-12-00941-s001.zip › S7.Figure6b50ugRb_r1.gif]

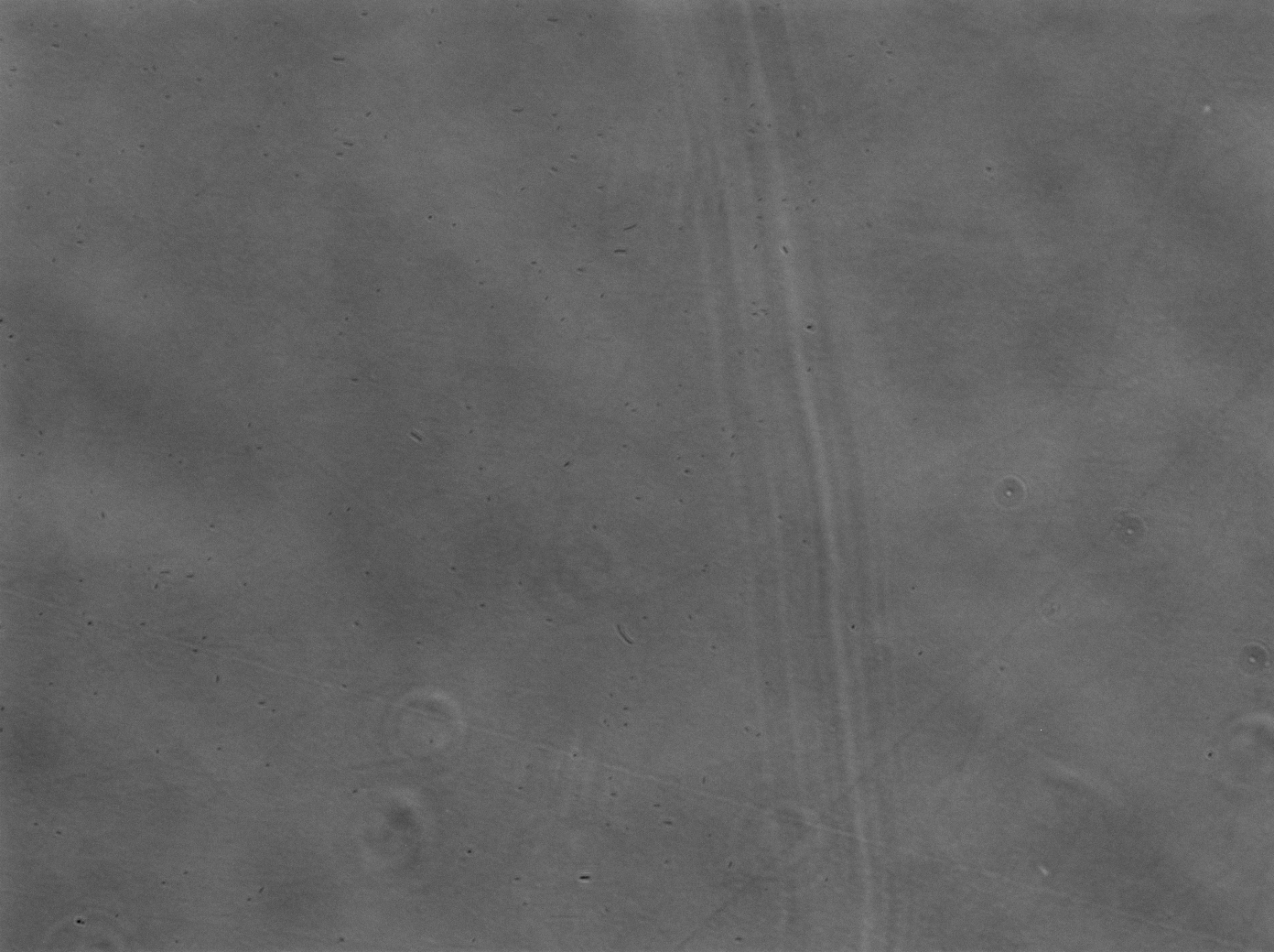

Supplement: Supplementary file 1 [file pathogens-12-00941-s001.zip › S8.Figure6b50ugRb_r2.gif]
